# Health-related Quality of Life in Vacuum-Assisted Breast Biopsy: short-term effects, long-term effects and predictors

**DOI:** 10.1186/1477-7525-8-11

**Published:** 2010-01-27

**Authors:** Philip J Domeyer, Theodoros N Sergentanis, Flora Zagouri, George C Zografos

**Affiliations:** 1 Breast Unit, First Department of Propaedeutic Surgery, Hippokratio Hospital, Medical School, University of Athens, 108 Vas Sofias Ave, Athens 11527, Greece

## Abstract

**Background:**

The impact of Vacuum-assisted breast biopsy (VABB, 11-Gauge) upon Health-related Quality of Life (HRQoL) remains an open field. This study aims to: i) assess short-term (4 days after VABB) responses in terms of HRQoL after VABB, ii) evaluate long-term (18 months after VABB) responses, if any, and iii) examine whether these responses are modified by a variety of possible predictors (anthropometric, sociodemographic, lifestyle habits, breast-related parameters, reproductive history, VABB-related features and complications, seasonality).

**Methods:**

This study included 102 eligible patients undergoing VABB and having benign lesions. A variable number of cores (24-96 cores) has been excised. HRQoL was assessed by EQ-5D and SF-36^® ^questionnaires: i) in the morning of the VABB procedure day (baseline measurement), ii) four days after VABB (early post-biopsy measurement) and iii) 18 months after VABB (late post-biopsy measurement). Statistical analysis comprised two steps: i. evaluation of differences in EQ-5D/SF-36 dimensions and calculated scores (baseline versus early post-biopsy measurement and baseline versus late post-biopsy measurement) and ii. assessment of predictors through multivariate linear, logistic, ordinal logistic regression, as appropriate.

**Results:**

At baseline patients presented with considerable anxiety (EQ-5D anxiety/depression dimension, EQ-5D TTO/VAS indices, SF-36 Mental Health dimension). At the early post-biopsy measurement women exhibited deterioration in Usual Activities (EQ-5D) and Role Functioning-Physical dimensions. At the late measurement women exhibited pain (EQ-5D pain/discomfort and SF-36 Bodily Pain), deterioration in Physical Functioning (SF-36 PF) and overall SF-36 Physical Component Scale (PCS). Mastalgia, older age and lower income emerged as significant predictors for baseline anxiety, whereas seasonality modified early activities-related responses. Pain seemed idiosyncratic.

**Conclusions:**

The HRQoL profile of patients suggests that VABB exerts effects prior to its performance at a psychological level, immediately after its performance at a functioning-physical level and entails long-term effects associated with pain.

## Background

Vacuum-Assisted Breast Biopsy (VABB) is a recently developed biopsy method, aiming to obtain tissue for histopathological diagnosis of non-palpable mammographic lesions. VABB can be performed under stereotactic or ultrasonographic guidance; an 11-Gauge (11G) needle is most commonly used for sampling of the suspicious lesion [1]. Although its role for sampling non-palpable breast lesions is already well established, the impact of VABB with 11-Gauge (11G) needle on health-related quality of life (HRQoL) has never been investigated.

We have already shown that psychological stress, which is an important aspect of HRQoL, is present before, during and after VABB, as depicted by the noteworthy increase in blood concentrations of stress hormones [2]. Furthermore, according to a study issued by our Unit, pain in women undergoing VABB is significant and follows an S-shape curve pattern; indeed the diameter of the needle emerged an important predictor of pain in different biopsy methods [3]. Apart from those short-term effects described by our team, mid-term (4 months after biopsy) effects of stereotactic breast biopsy have been recognized [4]. Indeed, according to our previous work, VABB seems to exhibit fairly distinct long-term effects, when compared to other biopsy methods in terms of compliance [5].

Given the above, it is rational to anticipate that VABB may exert significant effects upon HRQoL. Nevertheless, only two studies (COBRA study [6] and the study issued by Maxwell et al. [7]) have appeared assessing the impact of stereotactic core needle biopsy on HRQoL. It should be stressed however that the COBRA study had adopted a comparative approach (i.e. stereotactic 14G needle biopsy versus open breast biopsy) and focused exclusively on short-term responses, i.e. up to four days after biopsy. Similarly the study by Maxwell et al. has assessed the 14G setting covering a 30-day period after core biopsy [7].

As a result, short-term and long-term effects of VABB (11G) on HRQoL remain an open field. The particularities in VABB are worth investigating systematically, as the special features of VABB together with the larger (11G) needle diameter may exhibit a distinct HRQoL profile, as documented in the context of other phenomena such as pain [3]. Importantly, to our knowledge, no insight into predictors modifying the effect of VABB upon HRQoL has appeared in the literature.

This study aims to: i) assess short-term (4 days after VABB) responses in terms of HRQoL after VABB, ii) evaluate long-term (18 months after VABB) responses, if any, and iii) examine whether these responses are modified by a variety of possible predictors (anthropometric, sociodemographic, lifestyle habits, breast-related parameters, reproductive history, VABB-related features and complications, seasonality). To our knowledge, this is the first study to address these issues.

## Methods

### Patients

Exclusion criteria for this study were: previous breast cancer, severe comorbidity (psychiatric conditions, stroke, autoimmune diseases, cancer, severe coronary heart failure, i.e. NYHA stage III or IV). In addition patients diagnosed with precursor (atypical ductal hyperplasia, ADH and lobular neoplasia, LN) lesions, as well as carcinomas (ductal, in situ, DCIS or invasive, IDC, lobular carcinomas) were excluded from the study, as the follow-up/treatment of these conditions, respectively, may interfere with HRQoL measurements.

**Figure 1:**
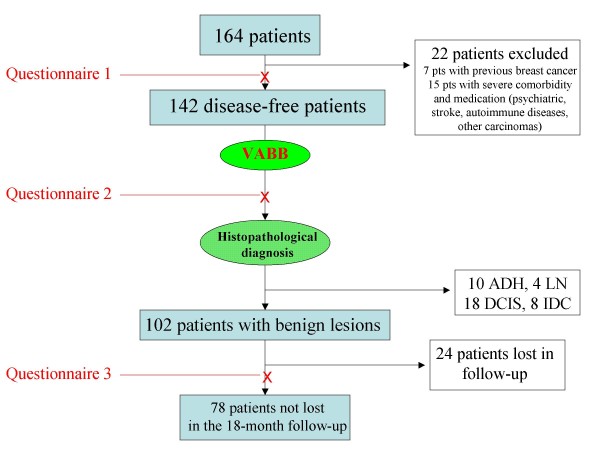
**Flow chart explaining the study design**.

Of the 164 consecutive patients who came to our Breast Unit due to non-palpable mammographic lesions requiring VABB, only 102 were eligible for this study (Figure 1). The women were 33-80 years old.

Patients were informed (orally and in written) about the procedure, possibility of pain and complications by the surgeon performing VABB. Written signed informed consent was obtained from all patients. The study was approved by the Local Institutional Review Board.

### VABB performance - local anesthesia

All patients presenting with a non-palpable mammographic lesion (microcalcifications, solid lesion or asymmetric density) BI-RADS 3 or 4 underwent VABB under stereotactic guidance (11G) on the Fisher's table (Mammotest, Fischer Imaging, Denver, CO, USA). According to the results of a double-blind study [8], a variable number of cores (24-96 cores) has been excised.

All procedures were performed by the same surgeon, in the same Unit, according to the recommended local anesthesia [1]; in addition two specialist radiologists assisted at the procedures. The surgeon performing VABB was familiar with this method before the onset of this study, having already performed 350 VABB procedures. For local anesthesia, the two-step approach was adopted: 5 cm^3 ^1% lidocaine without epinephrine (superficial) and 10 cm^3 ^1% lidocaine with epinephrine (deep) were administered. The biopsy was performed according to a standard protocol to assure quality control. Compression bandages were applied so as to prevent hematoma.

### HRQoL measurement

HRQoL was measured with the EQ-5D [9] and SF-36^® ^[10] questionnaires. EQ-5D encompasses five dimensions (mobility, self-care, usual activities, pain/discomfort and anxiety/depression), each one with three levels (no problems, some problems, extreme problems/unable). EQ-5D also contains a visual analogue scale on which patients rate their own health between 0 and 100 (designated as *EQ-5D VAS "thermometer"*) [9]. Based on patients' responses two indices were calculated: EQ-TTO (Time Trade-Off values) [11] and EQ-VAS [12]; the norms of the Spanish population were adopted under the light of geographical and social proximity. Importantly no Greek norms have been published to our knowledge.

The SF-36 questionnaire comprises 36 items covering eight health dimensions, namely physical functioning (PF), bodily pain (BP), general health (GH), vitality (VT), social functioning (SF), mental health (MH), role functioning-physical (RP) and role-functioning-emotional (RE). It produces a health profile with scores between 0 and 100 for each dimension [10]. Based on ratings two overall scores were calculated (Physical Component Scale, PCS and Mental Component Scale, MCS), once again using the Spanish norms [13].

### Structure and administration of questionnaires

All patients were asked to complete SF-36 and EQ-5D questionnaires simultaneously, i) in the morning of the VABB procedure day (i.e. 1-2 hours prior to biopsy, designated as *baseline measurement*), ii) four days after VABB (i.e. always prior to obtaining a final diagnosis of the breast lesion, designated as *early post-biopsy measurement*) and iii) 18 months after VABB (designated as *late post-biopsy measurement*).

At the baseline assessment the following information was obtained: i) anthropometric features (height, weight, from which Body Mass Index (BMI) was calculated), ii) sociodemographic parameters i.e. age, place of residence (urban or rural), education (1 = primary education, 2 = secondary education, 3 = technological educational institute, 4 = university, 5 = postgraduate university education), professional risk (0 = low risk, i.e. permanent employees and housewives, 1 = high risk, i.e. non-permanent job, for instance in the private sector or self-employed), marital status (married/living with partner, single, widowed, divorced), number of offspring (male and female separately), personal income, iii) lifestyle habits (current smoking), iv) breast-related parameters (mastalgia, presence of fibrocystic disease, breast cancer history in a first-degree relative, monthly breast self-examination, duration of breastfeeding), v) reproductive history (menopausal status, age at menarche, age at first full-term pregnancy, spontaneous abortions, miscarriages, number of prior caesarian sections, oral contraceptive/HRT (hormone replacement therapy) ever-use, vi) VABB-related features [referral, type of lesion (microcalcifications, solid lesion, asymmetric density), BI-RADS classification], vii) seasonality (biopsy month). Moreover, the volume of tissue excised, subsequent hematoma formation and infection were recorded after VABB. The histology of the lesion was classified according to the system first proposed by Dupont and Page [14] and adopted by the recent review by Guray and Sahin [15]. At the late post-biopsy measurement the satisfaction of patients with the cosmetic result was also recorded.

### Statistical analysis

**Figure 2:**
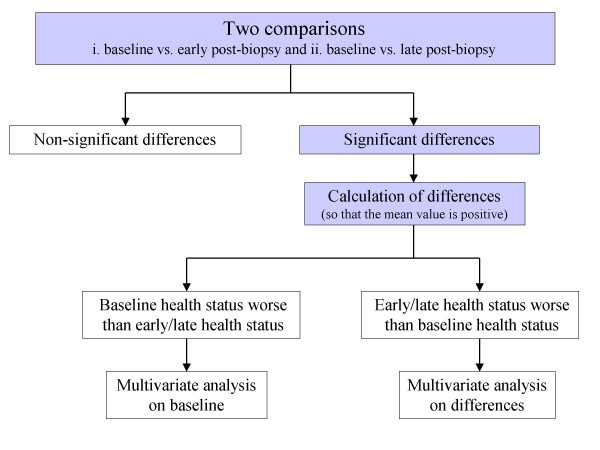
**Flow chart explaining the successive steps of the statistical analysis**.

Statistical analysis is summarized in Figure 2 and comprised two steps: 1. evaluation of differences in EQ-5D/SF-36 dimensions and calculated scores and 2. assessment of predictors. Concerning step 1, two comparisons were made: i. baseline versus early post-biopsy measurement and ii. baseline versus late post-biopsy measurement. Given that two comparisons were performed, the Bonferroni correction was adopted, i.e. the threshold for statistical significance was equal to 0.05/2 = 0.025.

Concerning step 2, the following procedure was followed: i. In case a difference was proven significant in step 1, the numerical difference was computed in such a way that the sign of the mean result was positive; for example, baseline minus post-biopsy difference was calculated for dimensions where mean baseline = mean post-biopsy value, whereas post-biopsy minus baseline difference was calculated for dimensions where mean post-biopsy = mean baseline value. This framework was adopted in order that the results be more tangible.

ii. After the calculation of differences two scenarios were possible: baseline health status was better or worse than subsequent (early/late) measurements.

It should be kept in mind that the point of focus of this study is the identification of predictors modifying the worsening (aggravation) of HRQoL at any time point before or after VABB. In an attempt to reach tangible and plausible results the design of the analysis also took into account the time criterion for causality.

Specifically: a) In case baseline values denoted worse health status, the multivariate analysis was performed on baseline values encompassing inherent features i.e. those acting prior to baseline. b) In case the subsequent measurements indicated worse health status than baseline, the analysis was performed on the calculated *differences*, encompassing inherent and VABB-related features as independent variables. The rationale underlying the setting of differences as dependent variables is the following: given the time criterion, some inherent possible predictors may have acted *both *at baseline and at subsequent measurements. However, as mentioned above, this study aims to examine whether predictors modify (further potentiate or limit) the aggravating effect of the procedure; as a result it is the change (gradient) that had to be modeled.

Concerning model building, the associations between baseline values or calculated differences and possible predictors were assessed first through univariate analysis; the predictors proven significant in the univariate analysis were included in the multivariate models. Where the assumptions of linear regression were met, the former was performed. When the assumptions of linear regressions were not met, the difference was converted to a binary variable (0 = values ≤ median, 1 = values above median). Concerning baseline measurements on EQ-5D dimensions ordinal logistic regression was performed.

The statistical analysis was performed using STATA 8.0 statistical software (Stata Corporation, College Station, TX, USA).

## Results

Table 1 outlines the features of the study sample; it is worth mentioning that no infections or hematomas requiring intervention were present in the study sample. The seven hematomas included in Table 1 are clinically significant hematomas with a diameter larger than 3 cm. Table 2 presents the changes in HRQoL in VABB. At the early measurement significant deterioration was noted in EQ-5D usual activities and SF-36 RP dimensions; on the other hand EQ-5D anxiety/depression dimension as well as EQ-5D indices (VAS and TTO) revealed worse health status at baseline.

**Table 1:** Description of the study sample (n = 102)

Categorical variables	Frequency (%)
***Sociodemographic parameters and lifestyle habits***	

Place of residence	
*Urban*	72 (70.6)
*Rural*	30 (29.4)

Education	
*Primary education*	21 (20.6)
*Secondary education*	44 (43.1)
*Technological educational institute*	10 (9.8)
*University*	22 (21.6)
*Postgraduate university education*	5 (4.9)

Professional risk	
*Low (permanent employees and housewives)*	68 (66.7)
*High (non-permanent job or self-employed)*	34 (33.3)

Marrital status	
*Married/living with partner*	84 (82.3)
*Single*	7 (6.9)
*Divorced*	5 (4.9)
*Widowed*	6 (5.9)

Current smoking	
*Yes*	27 (26.5)
*No*	75 (73.5)

***Breast-related parameters***	

Mastalgia	
*Yes*	30 (29.4)
*No*	72 (70.6)

Presence of fibrocystic disease	
*Yes*	47 (46.1)
*No*	55 (53.9)

Breast cancer history in a first-degree relative	
*Yes*	8 (7.8)
*No*	94 (92.2)

***Reproductive history***	
Menopausal status	
*Premenopausal*	43 (42.2)
*Postmenopausal*	59 (57.8)

Number of prior caesarian sections	
*None*	86 (84.3)
*One*	7 (6.9)
*Two*	7 (6.9)
*Three*	2 (1.9)

***VABB-related features and histological classification***	

Histological classification	
*Nonproliferative lesions*	
*Mild epithelial hyperplasia*	9 (8.8)
*Ductal ectasia*	5 (4.9)
*Nonsclerosing adenosis*	1 (1.0)
*Periductal fibrosis*	4 (3.9)
*Multiple coexisting nonproliferative lesions*	15 (14.7)
*Proliferative lesions*	
*Moderate ductal hyperplasia without atypia*	15 (14.7)
*Sclerosing adenosis*	7 (6.9)
*Radial scar*	1 (1.0)
*Intraductal papilloma*	5 (4.9)
*Intraductal papillomatosis*	1 (1.0)
*Multiple coexisting proliferative lesions*	13 (12.7)
*Fat necrosis*	1 (1.0)
*Fibroadenomas*	
*Without coexisting lesions*	11 (10.8)
*With coexisting nonproliferative lesions*	8 (7.8)
*With coexisting proliferative lesions*	6 (5.9)

BI-RADS classification	
*BI-RADS 3*	36 (35.3)
*BI-RADS 4*	66 (64.7)

Hematoma	
*Yes*	7 (6.9)
*No*	95 (93.1)

Biopsy season	
*Spring*	28 (27.5)
*Summer*	26 (25.4)
*Autumn*	31 (30.4)
*Winter*	17 (16.7)

**Continuous variables**	**Mean ± SD (median)**

Age (years)	51.3 ± 8.8 (50.5)

BMI (kg/m^2^)	25.4 ± 3.9 (24.8)

Personal income (euro)	870 ± 860 (735)

Number of offspring	1.8 ± 1.0 (2.0)

Volume of tissue excised (cc)	4.0 ± 3.2 (3.0)

**Table 2:** Baseline, early and late post-biopsy HRQoL measurements

Variables	Baseline (mean ± SD)	Early post-biopsy measurement (mean ± SD)	*p*§	Late post-biopsy measurement (mean ± SD)	*p *†	Practical interpretation
**EQ-5D dimensions and indices**						

Mobility*	1.41 ± 0.49	1.42 ± 0.50	0.782	1.27 ± 0.45	**0.011**	Deterioration at baseline

Self-care*	1.03 ± 0.17	1.03 ± 0.17	1.000	1.09 ± 0.33	0.058	No changes

Usual activities*	1.24 ± 0.43	1.31 ± 0.46	**0.021**	1.23 ± 0.45	0.835	Short-term deterioration

Pain/discomfort*	1.53 ± 0.59	1.55 ± 0.61	0.977	1.71 ± 0.54	**0.004**	Long-term deterioration

Anxiety/depression*	1.98 ± 0.65	1.52 ± 0.61	**<0.0001**	1.77 ± 0.60	**0.002**	Deterioration at baseline

VAS "thermometer"	68.8 ± 18.4	67.5 ± 18.1	0.406	75.5 ± 15.6	**0.003**	Deterioration at baseline

EQ-5D index *(TTO method)*	0.729 ± 0.224	0.787 ± 0.208	**0.005**	0.769 ± 0.225	0.251	Deterioration at baseline

EQ-5D index *(VAS method)*	0.834 ± 0.076	0.854 ± 0.062	**0.038**	0.845 ± 0.085	0.324	Deterioration at baseline

						

**SF-36 dimensions and scores**						

Physical functioning	86.2 ± 19.5	85.2 ± 18.9	0.641	80.1 ± 19.4	**0.0001**	Long-term deterioration

Bodily pain	78.3 ± 26.4	76.3 ± 27.5	0.414	65.5 ± 30.5	**0.0004**	Long-term deterioration

General Health	64.5 ± 21.3	68.5 ± 22.5	0.067	65.6 ± 19.0	0.700	No changes

Vitality	60.6 ± 19.5	60.8 ± 18.7	0.999	59.6 ± 21.9	0.697	No changes

Social Functioning	75.3 ± 24.7	74.6 ± 25.3	0.496	73.4 ± 27.6	0.925	No changes

Mental Health	58.8 ± 19.3	60.4 ± 20.2	0.139	62.8 ± 21.1	**0.030**	Deterioration at baseline

Role functioning-physical	80.1 ± 33.1	72.3 ± 39.2	**0.008**	73.4 ± 39.0	0.098	Short-term deterioration

Role functioning-emotional	71.0 ± 37.0	70.9 ± 37.1	0.650	66.2 ± 42.1	0.347	No changes

Physical Component Scale	52.5 ± 8.6	51.8 ± 7.9	0.234	48.5 ± 9.3	**0.004**	Long-term deterioration

Mental Component Scale	40.1 ± 11.8	41.5 ± 11.6	0.270	41.9 ± 14.3	0.568	No changes

§ p-values derived from Wilcoxon matched-pairs signed-ranks test (early post-biopsy measurement vs. baseline)

†: p-values derived from Wilcoxon matched-pairs signed-ranks test (late post-biopsy measurement vs. baseline)

*: Measures where a higher score denotes a worse health status

Concerning the baseline-late post-biopsy comparison, worse health status in the late measurement was demonstrated through EQ-5D pain/discomfort dimension, SF-36 PF and BP dimensions as well as SF-36 PCS overall score; on the contrary baseline denoted worse health state in EQ-5D mobility, anxiety/depression, VAS "thermometer" measurements as well as SF-36 MH dimension.

Table 3 presents predictors assessed through the baseline-early post-biopsy comparison. Biopsy season was associated with more pronounced worsening in EQ-5D usual activities dimension; on the contrary greater number of prior cesarean sections was associated with less pronounced worsening in SF-36 RP dimension. Regarding the dimensions pointing to worse status in baseline, mastalgia was associated with higher degree of anxiety/depression and, consequently, worse health status as measured by EQ-5D TTO and VAS indices. Increasing age was associated with worse baseline EQ-5D TTO and VAS indices; on the other hand increasing income correlated with better baseline EQ-5D TTO values.

**Table 3:** Predictors emerging through the assessment of baseline vs. early post-biopsy measurement

Dimensions where **early** post-biopsy measurement denoted **worse** health status than baseline			
**Dimensions/scores**	**Category or increment**	**OR or Coeff**. § **(95%CI)**	**p**

EQ-5D Usual activities		OR (95% CI)	
*Biopsy season*	*summer→autumn/spring→winter*	8.65 (1.99-37.51)	0.004

SF-36 RP		Coeff. (95% CI)	
*Number of prior caesarean sections*	*1 procedure increase*	-12.3 (-24.8, +0.2)	0.053

Dimensions where **baseline **measurement denoted **worse **health status than early post-biopsy*			

EQ-5D Anxiety/depression		OR (95% CI)	
*Mastalgia*	*yes vs no*	3.22 (1.11-9.35)	0.032

EQ-5D index (TTO)		Coeff. (95% CI)	
*Age*	*10 year increase*	-0.06 (-0.12, -0.01)	0.025
*Personal income*	*100 euro increase*	0.008 (-0.0004, 0.016)	0.061
*Mastalgia*	*yes vs no*	-0.16 (-0.27, -0.04)	0.010

EQ-5D index (VAS)		Coeff. (95% CI)	
*Age*	*10 year increase*	-0.02 (-0.04, -0.003)	0.014
*Mastalgia*	*yes vs no*	-0.05 (-0.09, -0.01)	0.017

* The analysis was performed on baseline values

§Coeff. was yielded from linear regression, OR (odds ratio) was derived from logistic regression in the case of EQ-5D Usual activities and from ordinal logistic regression in the case of EQ-5D Anxiety/depression.

Table 4 presents predictors assessed through the baseline-late post-biopsy comparison. No significant predictors were found for the worsening noted in EQ-5D pain/discomfort and SF-36 BP dimensions. Mastalgia was associated with more marked deterioration in SF-36 PF dimension and PCS overall score; interestingly current smoking and being married seemed to play a protective role for SF-36 PF and SF-36 PCS deterioration, respectively. Concerning the dimensions suggesting worse status at baseline, age was associated with worse EQ-5D mobility status and worse EQ-5D VAS "thermometer" values; similarly mastalgia unfavorably modified EQ-5D VAS "thermometer" and SF-36 MH. Personal income predicted better health status as measured by EQ-5D mobility dimension.

**Table 4:** Predictors emerging through the assessment of baseline vs. late post-biopsy measurement

Dimensions where **late** post-biopsy measurement denoted **worse** health status than baseline			
**Dimensions/scores**	**Category or increment**	**OR or Coeff**. § **(95% CI)**	**p**

EQ-5D Pain/discomfort		No significant predictors found	

SF-36 Physical Functioning		Coeff. (95% CI)	
*Mastalgia*	*yes vs no*	13.4 (5.1, 21.8)	0.002
*Current smoking*	*yes vs no*	-8.1 (-16.6, +0.5)	0.063

SF-36 Bodily Pain		No significant predictors found	

SF-36 Physical Component Scale		Coeff. (95% CI)	
*Mastalgia*	*yes vs no*	8.6 (2.2, 15.1)	0.010
*Marital status*	*married vs single/divorced/widowed*	-9.4 (-16.5, -2.3)	0.010

Dimensions where **baseline **measurement denoted **worse **health status than late post-biopsy*			

EQ-5D Mobility		OR (95% CI)	
*Age*	*10 year increase*	1.81 (1.00-3.28)	0.051
*Personal income*	*100 euro increase*	0.88 (0.80-0.95)	0.002

EQ-5D Anxiety/depression *Mastalgia*	See Table 3		

EQ-5D VAS "thermometer"		Coeff. (95% CI)	
*Age*	*10 year increase*	-8.0 (-12.6, -3.5)	0.001
*Mastalgia*	*yes vs no*	-13.6 (-23.2, -4.1)	0.006

SF-36 Mental Health		Coeff. (95% CI)	
*Mastalgia*	*yes vs no*	-20.0 (-29.5, -10.5)	<0.001

* The analysis was performed on baseline values

§Coeff. was yielded from linear regression, OR (odds ratio) was derived from logistic regression in the case of EQ-5D Mobility.

Patients were satisfied with the cosmetic result (75/78, 96.2%); satisfaction with the cosmetic result was not associated with any HRQoL measurement. Noticeably the histology of lesions was not associated with any HRQoL measurement.

## Discussion

**Figure 3:**
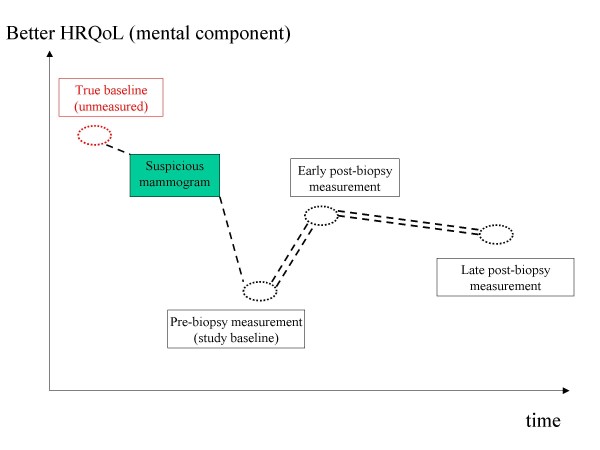
**Theoretical framework explaining the anxiety demonstrated prior to VABB**. Double-dashed lines indicate the phases included in the study (baseline, early post-biopsy and late post-biopsy).

This study is the first to document that VABB is capable of modifying HRQoL in a multifaceted, complex way. Interestingly enough, the effects of VABB upon HRQoL seem to have begun well before the biopsy procedure *per se*. Strikingly, patients' anxiety prior to biopsy is so considerable that it led to significantly worse overall (VAS and TTO) HRQol EQ-5D indices when compared to the early post-biopsy measurement. As a result, a pattern emerges, according to which women come to the biopsy procedure with already aggravated HRQoL (Figure 3). The existence of this phenomenon is methodologically and conceptually challenging, as the true baseline remains elusive, being located prior to the suspicious mammogram. It is worth mentioning that our result are in accordance with previously published studies, which have documented significant anxiety prior to other methods of breast biopsy [7,16]. As a result awaiting a biopsy for a potential malignancy emerges as a factor capable of creating anxiety irrespective of the method of biopsy.

Apart from the above finding, VABB was capable of generating substantial short- and long-term effects upon subjects' HRQoL. At the early measurement, a limitation of the capability to perform usual activities (EQ-5D) and deterioration of the SF-36 Role Functioning-Physical scale point to pain and discomfort after the procedure. Interestingly enough, this study points additionally to long-term pain after VABB, as reflected upon the directly relevant late measurements of EQ-5D pain/discomfort scale and the SF-36 Bodily Pain dimensions, as well as possibly upon the SF-36 Physical Component Scale overall score. To our knowledge, this is the first time that such an observation is reported in the literature. Long-term effects of VABB, such as scar formation [17,18], have been reported, especially in the context of greater tissue amount excised [17]. Whether the underlying, scar formation-related distortions of breast architecture together with inflammatory phenomena may be accompanied by long-term pain is an issue that has never before been addressed.

Making one step beyond the demonstration of significant changes, this study has investigated the existence of predictors capable of modifying the responses of women in terms of HRQoL before and after VABB. The predictors may be schematically divided into those affecting the baseline, mainly anxiety-related, status and those affecting subsequent, early or late, responses.

Concerning baseline, mastalgia emerged as a particular risk factor for anxiety, acting unfavorably upon EQ-5D anxiety/depression dimension, EQ-5D thermometer, EQ-5D overall VAS and TTO indices, as well as SF-36 Mental Health dimension. It seems fairly rational to postulate that women who have experienced mastalgia are more concerned about their breast health and thus present with more pronounced anxiety. In addition, mastalgia has been associated with a host of conditions including mood disorders, post-traumatic stress disorder, eating disorders and pain-related conditions [19], which may interfere with measurements of anxiety. Apart from mastalgia, lower income and older age were risk factors for worse HRQoL prior to biopsy, rather expectably.

Regarding early effects of VABB, biopsy season was a risk factor for worsening in EQ-5D usual activities scale. This may be explainable, if the bulk of subjects' everyday, usual activities is taken into account; usual activities are more demanding in winter, compared to the lower pace in summer. It is worth mentioning that seasonality may not be safely extrapolated to other cultures or countries, as this effect of summer may represent a Greek or Mediterranean particularity. Concerning early effects, it is also worth reporting that prior caesarian sections were associated with less pronounced deterioration in SF-36 Role Functioning-Physical scale, suggesting that women who have undergone previous gynecological surgery seem more "resistant" to early unfavorable effects of VABB; in other words, women with prior caesarian sections may be accustomed to temporary or short-term pain.

Commenting on late effects, a striking finding is that long-term pain (EQ-5D pain/discomfort and SF-36 Bodily Pain dimensions) seemed rather idiosyncratic, since none of the predictors examined, including the volume of tissue excised, was proven significant. One possible explanation of this observation may be the fact that sampling was performed at the "higher limits" i.e. above 24 cores; as a result the threshold of significant pain might already have been reached at 24 cores. Another explanation might essentially entail breast size as a confounder, i.e. background correlation between larger number of excised cores and larger breast size. Although breast size was not included in the study design and in thus unavailable, it should be declared that the consecutive cases in this study have been derived from a wider pool of patients 1:1 randomly allocated to 24 and 96 cores (i.e. extension of our double-blind study [8]). As a result the effect of unknown confounders such as breast size should be considered minimal, if any. Nevertheless, future studies stratifying results would be of interest so as to gain more detailed insight into the phenomenon of long-term pain.

Further commenting on late effects, once again mastalgia emerged as an unfavorable risk factor, being associated with more pronounced deterioration in SF-36 Physical Functioning dimension and overall SF-36 Physical Component Scale. Surprisingly enough, smoking emerged as a favorable factor, limiting the deterioration in SF-36 Physical Functioning dimension; it is tempting to attribute this finding to analgesic and stress-modulating effects of nicotine (reviewed in [20]). Another favorable factor is marital status, as married patients displayed a better profile in SF-36 Physical Component Scale overall score; this may reflect the supportive role of the partner.

This study, however, bears certain limitations that should be addressed. Firstly, some features of our setting need to be clarified. In our study, VABB was exclusively performed under stereotactic guidance. Therefore, the results may not be extrapolated to ultrasound-guided VABB. Indeed, given that one of the major complaints of patients undergoing stereotactically-guided VABB is the discomfort experienced in a prone position [5], ultrasound-guided VABB might be better tolerated and might consequently exhibit a different pattern of early effects upon HRQoL. Envisaging comparative studies assessing VABB (stereotactically- vs ultrasound-guided) or even encompassing other biopsy procedures, e.g. core biopsy, would be promising; however the present study has not adopted a comparative study design leaving the field open for future studies.

In addition, the fact that all biopsy procedures have been performed by a surgeon does not obligatorily reflect breast radiologists' practice; this may be a significant limitation which should be born in mind for the extrapolation of these findings to other settings. Nevertheless the exact nature of differences between surgeons' and radiologists' practice remains to be elucidated in future comparative studies. An additional limitation which should be considered prior to any efforts of extrapolation is the number of cores excised in our setting; 24-96 cores represent a relatively large volume of tissue removed in comparison to other settings [8]. Moreover, the proportion of women lost in follow-up (24/102) might represent a limitation, as optimal compliance to follow-up would be desirable. Furthermore, a limitation pertaining to analgesia [21] is worth addressing; although analgesia was not prescribed to any patient, the potential over-the-counter use of paracetamol cannot be excluded. An additional limitation is the fact that no classification of mastalgia was adopted (cyclic, noncyclic). Nevertheless, this study points to the need for further studies assessing the impact of specific features of mastalgia upon HRQoL.

An important limitation concerning the analysis of data should be acknowledged. Mixed-effects models represent the optimal solution for longitudinal data; however, given our relatively small sample size, the necessary number of variables and interactions (for the simultaneous assessment of time trends and modifying effects of inherent clinical variables) would render the implementation of such models not robust enough. Consequently we had to proceed to separate Generalized Linear Models analyses, as presented above.

## Conclusions

The HRQoL profile of patients suggests that VABB exerts effects prior to its performance at a psychological level, immediately after its performance at a functioning-physical level and entails long-term effects associated with pain. Mastalgia, older age and lower income emerged as significant predictors for baseline anxiety, whereas seasonality modified early activities-related responses. Pain seemed idiosyncratic.

## Competing interests

The authors declare that they have no competing interests.

## Authors' contributions

PJD conceived the idea of the study, designed the study, acquired data, performed statistical analysis, interpreted data in the context of the international literature and drafted the manuscript. TNS designed the study, performed statistical analysis, interpreted data and drafted the manuscript. FZ acquired data, interpreted data and revised the manuscript critically for important intellectual content. GCZ designed the study, performed VABB, revised the manuscript for important intellectual content and gave final approval of the version to be published.
